# Towards imaging the infant brain at play

**DOI:** 10.1080/19420889.2023.2206204

**Published:** 2023-05-09

**Authors:** Aleksandra A. W. Dopierala, Lauren L. Emberson

**Affiliations:** Baby Learning Lab, Department of Psychology, University of British Columbia, Vancouver, BC, Canada

**Keywords:** infant, neuroimaging, fNIRS, developmental psychology, naturalistic design, neurocognitive development, object exploration

## Abstract

Infants’ first-person experiences are crucial to early cognitive and neural development. To a vast extent, these early experiences involve play, which in infancy takes the form of object exploration. While at the behavioral level infant play has been studied both using specific tasks and in naturalistic scenarios, the neural correlates of object exploration have largely been studied in highly controlled task settings. These neuroimaging studies did not tap into the complexities of everyday play and what makes object exploration so important for development. Here, we review selected infant neuroimaging studies, spanning from typical, highly controlled screen-based studies on object perception to more naturalistic designs and argue for the importance of studying the neural correlates of key behaviors such as object exploration and language comprehension in naturalistic settings. We suggest that the advances in technology and analytic approaches allow measuring the infant brain at play with the use of functional near-infrared spectroscopy (fNIRS). Naturalistic fNIRS studies offer a new and exciting avenue to studying infant neurocognitive development in a way that will draw us away from our laboratory constructs and into an infant’s everyday experiences that support their development.

Infants’ first-person experiences are crucial to early cognitive and neural development. To a vast extent, these early experiences involve play, which in infancy takes the form of object exploration. Across the world, infants engage with objects every day (e.g [1]. [2]; and object play is a universal context for learning [3]. Indeed, early object exploration has been linked with future cognitive outcomes. For instance, experience with sticky mittens – special mittens that allow infants to pick up objects before they learn to grasp – increases infants’ object engagement [4]; the amount of object exploration across infancy relates to language, motor, and cognitive development throughout toddlerhood [5]. While at the behavioral level infant play has been studied both using specific tasks (e.g., sticky mittens) and in naturalistic scenarios (e.g., free play [6], the neural correlates of object exploration have largely been studied in highly controlled task settings.

In most neuroimaging studies, infants are seated in front of a puppet show or a screen and presented with a series of objects, differing in shapes and colors, while their brain responses are recorded. These studies shed light on the development of neural correlates of object exploration showing that several brain regions involved in object perception in adults are functionally active already in infancy. For instance, the anterior temporal cortex, involved in higher-level object processing in adults (e.g [7], responded when infants differentiated the shape of the objects at 3 months of age [8]; The lateral occipital cortex (LOC), a region involved in object processing and shape perception in adults [9,10], showed similar functional specialization by 6 months of age [11].

While these studies revealed the neural correlates of object processing in the infant’s brain, they did not tap into the complexities of everyday play and what makes object exploration so important for development. Firstly, we know that infant play involves more than passively looking at objects – infants manipulate the objects, put them in their mouth, and drop them to the floor. Secondly, infant play is embedded in the sociocultural context of the environment. Infants explore objects during interactions with caregivers, who talk about and act on the objects [12]. Thirdly, these interactions differ between cultures [13,14] and thus are not well reflected by the standardized tasks used in the neuroimaging literature. Finally, there’s research showing that infant performance in laboratory tasks does not directly translate to everyday experiences e.g [15].

Thanks to the advances in technology and analytic approaches [16–18], measuring the infant brain at play is now possible. A recent shift in developmental cognitive neuroscience sees moving away from the standard and toward more naturalistic studies. One technology is particularly promising for imaging the infant brain at play: functional near-infrared spectroscopy (fNIRS, for a review see [19]. FNIRS is a noninvasive method that uses light to detect changes in the concentration of hemoglobin in the cortex. Thus, similarly to fMRI, fNIRS provides an indirect measure of brain functional activity based on blood oxygenation levels. Unlike fMRI, fNIRS systems consist of lightweight headgear that can be comfortably worn for long periods and allows participants to move freely.

Supported by this technology, fNIRS researchers are embracing increasingly naturalistic paradigms. Considering the last 10–15 years, studies have had infants watch the experimenter perform specific actions instead of watching prerecorded videos [20] or interact with the experimenter [21–24]. While such experiments were more naturalistic – as they involve a live presenter – they did not reflect the infant’s daily experience. Unlike the everyday play, they offer a highly structured experience (i.e., the use of precisely timed periods of interaction and “rest” such as experimenter looking at the infant for 10 secs and then looking away from the baby for a further 10 sec and alternating), and no space for the infant to play an active role in this engagement. An alternative to such naturalistic but structured tasks is the free play paradigm, where caregivers engage infants in play just as they typically would at home. A few fNIRS studies already measured the infant brain during free play [25; 26], for a review see [27]. Bhat and colleagues measured infant brain responses during object exploration and interaction with the parent. They found that infants at risk of ASD showed reduced activation compared to low-risk infants. Nguyen and colleagues measured interpersonal neural synchrony while infants and caregivers watched something together or engaged in face-to-face play (no toys). They found that neural synchrony between the infant and the mother was highest during play, likely stemming from the presence of social touch. Thus, fNIRS research is becoming increasingly naturalistic allowing researchers to get closer to investigate the neural mechanisms supporting object play in infancy.

But truly naturalistic studies will allow us to dive into the complexity of infants’ first-person experiences. As we know from behavioral studies already, free play is so much more than just object exploration: infants vocalize, touch their own bodies, look and listen to the caregiver’s speech (including object labels), and singing. Naturalistic fNIRS studies using the free play paradigm will record neural responses to all of these experiences and behaviors and, therefore, will give us a window into the complexities of infant first-person experiences. Future studies will be able to look into the influence of labeling on infants’ brain responses to object exploration, how different types of object exploration (manual vs. visual vs. oral) influence neural engagement as well as how these behaviors and their neural correlates vary across different socio-cultural contexts. Finally, naturalistic fNIRS studies offer a new and exciting avenue to studying infant neurocognitive development in a way that will draw us away from our laboratory constructs and into an infant’s everyday experiences that support their development.
